# Determinants of mortality of juvenile harbour seals (*Phoca vitulina*) infected with lungworm submitted to a Dutch seal rehabilitation centre

**DOI:** 10.1016/j.ijppaw.2020.12.002

**Published:** 2020-12-13

**Authors:** M.F.A. van Wijngaarden, M.I.M. Geut, J.C.M. Vernooij, L.L. IJsseldijk, T.J. Tobias

**Affiliations:** a Utrecht University, Faculty of Veterinary Medicine, Department of Population Health Sciences, Yalelaan 7, 3584 CL, Utrecht, the Netherlands; b A Seal - Seal Rescue and Rehabilitation Centre Stellendam, Haringvlietplein 3A, 3251 LD, Stellendam, the Netherlands; c Utrecht University, Faculty of Veterinary Medicine, Department of Biomolecular Health Sciences, Division of Pathology, Yalelaan 7, 3584 CL, Utrecht, the Netherlands

**Keywords:** *Phoca vitulina*, Grey seal, *Halichoerus grypus*, *Otostrongylus circumlitus*, *Parafilaroides gymnurus*, Survival, Epidemiology

## Abstract

Since the seal populations in the North Sea are again thriving, the rationale behind seal rehabilitation is currently under discussion. Seals frequently require rehabilitation as a result of a lungworm infection, with these infections most commonly seen in young seals. The need for triage support is addressed by the organisations involved in seal rehabilitation to ensure adequate decision making on whether or not a seal should be taken into rehabilitation. It is still unclear which parameters influence seal mortality in rehabilitation, these parameters are essential to enable triaging of stranded seals.

Therefore, the aims of this study were: to estimate the proportion of lungworm infected juvenile harbour seals in a rehabilitation centre; to determine the survival rate among lungworm infected juvenile harbour seals; and to study determinants of mortality in the lungworm infected juvenile harbour seals. Data was collected retrospectively from all harbour seals admitted to a Dutch rehabilitation centre between September 2017 and August 2019 (n = 208). Eleven parameters were evaluated using univariable logistic regression with a 95% confidence interval (p < 0.05) to study the association between the determinants and the outcome – survival or death. All associated parameters with a p-value <0.2 were used in multivariable logistic regression.

The multivariable model demonstrated that high body temperature at intake (high vs normal body temperature OR = 0.32; p = 0.01); intake from August to December (Augustus-December vs January–May OR = 0.40; p = 0.02); and whether the seal was previously admitted to a rehabilitation centre (yes vs no OR = 0.12, p < 0.01) were good determinants of mortality. The results of this study could be used to further develop triage-support that aids in the decision to leave the seal on the beach; admitting the seal to a rehabilitation centre; and/or to euthanise the seal, in order to prevent further suffering.

## Highlights

• First study of determinants of mortality in juvenile harbour seals with lungworm.
• 91.8% of rehabilitated juvenile harbour seals are treated for lungworm.
• The mortality in lungworm infected rehabilitated juvenile harbour seals is 35.3%.
• Determinants for mortality are body temperature; previously rehabilitated; and period of the year.
• Outcomes could be used to assist in triage of stranded seals.

## Graphical abstract

**Figure:**
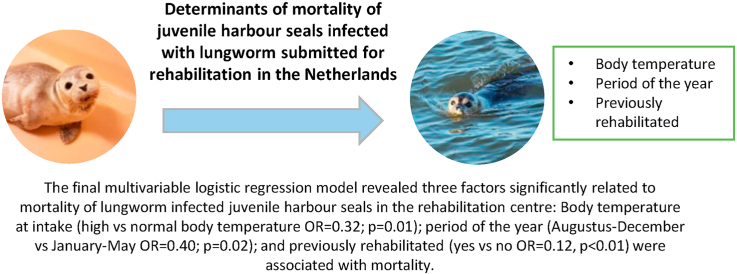

## 1 Introduction

Two seal species live in the North Sea surrounding the Netherlands: harbour seals (*Phoca vitulina)* and grey seals (*Halichoerus grypus*) (Hall and Russell, 2018; Teilman and Galatius, 2018). In 2016, approximately 14,100 seals – 9000 harbour seals and 5100 grey seals - resided in the Dutch North Sea (Van der Zande et al., 2018). In the same year, approximately 450 seals were rehabilitated in Dutch seal rehabilitation centres (Brasseur, 2018). Seals frequently require rehabilitation as a result of lungworm infection, with these infections most commonly seen in young seals (Van der Zande et al., 2018). Ulrich et al. (2016), investigated lungworm seroprevalence of wild harbour seals in the North Sea, discovering that the prevalence in wild juvenile harbour seals was 89%.

Seals are mostly infected with one or two types of lungworm (metastrongyloid) species: *Otostrongylus circumlitus* and/or *Parafilaroides gymnurus* (Railliet, 1899; Barnett and Bexton, 2016). *Otostrongylus circumlitus* are large bronchial worms (♂ = 53.3 ± 3.5 mm; ♀ = 62.0 ± 11.0 mm) (Leidenberger and Böstrom, 2009; Measures, 2018), that can cause obstructive bronchitis and bronchiolitis (Field et al., 2018). During their migration to the lungs, *O. circumlitus* are sometimes also found in the pulmonary artery, the right ventricle of the heart, the lymph nodes or the liver (Measures, 2018). Young seals (<1 year old) are primarily susceptible to infections with *O. circumlitus*, as a result of either incomplete immunity or having a different diet from (sub)adult seals (Measures, 2018). Infections with *O. circumlitus* can result in several lesions in the lungs, including bronchitis, bronchopneumonia, areas of pulmonary haemorrhage and pulmonary arteritis (Measures, 2018). Secondary bacterial infections are common. *Parafilaroides gymnurus* are small lungworms (16.23 ± 3.30 mm) (Osinga, 2015). Adult *P. gymnurus* are mostly present in the alveoli and in the small bronchioles of phocids (Field et al., 2018; Measures, 2018). The pathogenesis of *Parafilaroides* spp. has not been extensively studied, but research on either stranded or hunted seals showed various lesions, such as: mild inflammation around firm granulomatous nodules (Onderka, 1989), localised haemorrhage (Measures, 2001), pulmonary oedema, bronchopneumonia and abscess formation (with secondary bacterial infections) (Measures, 2018). Adult *P. gymnurus* can sometimes cause acute bronchitis and bronchopneumonia (Measures, 2001). Incidentally, *P. gymnurus* are also found in the pulmonary arteries (Measures, 2018).

The extent of lungworm infection and the associated inflammatory response vary significantly among seals. Some seals have no inflammatory response and present no clinical signs of infection, while other seals suffer from marked suppurative granulomatous pneumonia (Field et al., 2018). Treatment of lungworm affected seals in rehabilitation centres generally consists of anti-helminthics i.e. fenbendazole and/or ivermectin per os or per subcutaneous injection. In addition, supportive therapy is often required, such as administration of corticosteroids or nonsteroidal anti-inflammatory drugs (NSAIDs) to limit the inflammatory reaction to the deceased lungworms (Barnett and Bexton, 2016; Field et al., 2018). The inflammatory response to deceased lungworms is often more severe than that to live lungworms (Field et al., 2018). Furthermore, treatment with antibiotics is occasionally applied for control and treatment of secondary bacterial infections (Barnett and Bexton, 2016).

The necessity of rehabilitating seals is being discussed, as the seal populations in the North Sea are thriving (Bowen, 2016; Lowry, 2016). A scientific advisory committee (SAC) on seal rehabilitation, initiated by the Dutch government in 2018, specifically emphasised that the number of lungworm infected harbour seals admitted to rehabilitation centres should be reduced (Van der Zande et al., 2018). The SAC stated that seal rehabilitation potentially interferes with natural selection due to lungworm infection in the wild population (Van der Zande et al., 2018). Assessment of lungworm associated disease and determining prognosis is difficult as pinnipeds are capable of masking severe disease (Field et al., 2018) and clinical signs in seals infected with lungworm are variable. Hence, it is necessary to develop a method of triaging stranded seals that aids in the decision to leave the seal on the beach, transport it to a rehabilitation centre and/or to euthanise the seal to prevent further suffering. Therefore, the aims of this study were: 1) to estimate the proportion of lungworm infected juvenile harbour seals in a rehabilitation centre; 2) to determine the survival rate of the lungworm infected juvenile harbour seals; and 3) to study determinants of mortality in the lungworm infected juvenile harbour seals.

## 2 Materials and methods

A cross sectional study was conducted, using records of rehabilitated seals from ‘A Seal’, a seal rehabilitation centre in the Netherlands. A Seal takes in stranded seals from a ±150 km long defined coastal area of the North Sea, from the Belgian border to the Dutch city of IJmuiden. Each year, 100–200 seals are admitted to the rehabilitation centre.

### 2.1 Data collection and preparation

The data was collected from the rehabilitated seals’ electronic records. All harbour seals which were admitted to the rehabilitation centre from September 1, 2017 to August 31, 2019 (n = 208) were included in the dataset. The majority of clinical data used for analysis was derived from standardised clinical examination at intake. Additionally, data on lungworm diagnosis and treatment was collected.

The following data was collected at intake:

• *Period of the year*; categorised into two groups: seals that were admitted to the rehabilitation centre from August to December and from January to May.
• *Weight (kg);* to get an indication of its body condition score.
• *Sex*; determined by the anogenital distance: <1 cm for females and >10 cm for males.
• *Body temperature* (°C); the rectal body temperature was measured with a conventional thermometer (Microlife VT 1831). Reference values for body temperature of captured seals are not available in the literature. In the rehabilitation centre the range 36.5–37.9 °C is considered as normal and therefore seals were categorised into three groups: seals with a high body temperature (>37.9 °C), seals with a normal body temperature (36.5–37.9 °C) and seals with a low body temperature (<36.5 °C).
• *Presence of ID tag or chip;* to determine if the seal had been previously admitted to a rehabilitation centre the hind flippers were checked for the presence of an identification tag from a rehabilitation centre. Additionally, the seal was checked for the presence of an identification chip using the Virbac BackHome ISO MAX V® chip reader. All licensed Seal rehabilitation centres in the Netherlands apply an ID tag to any seal that is rehabilitated, containing an individual ID number and a way to identify the previous rehabilitation centre (either colour or full name and/or address).
• *Standard length and axillary girth (cm)*; standard length was measured from the nose to the tip of tail (nose-tail length). Additionally, a seal was measured from the nose to the edge of the hind flipper (nose-flipper length). Axillary girth was measured directly behind the front flippers.
• *Body condition score;* two body condition scoring methods were used. The first body condition score (I) is calculated using body weight and standard length (nose-tail length): Body condition score I = body weight/standard length * 100 (Noren et al., 2014). The second body condition score (II) is calculated using axillary girth and standard length (nose-tail length): Body condition score II = axillary girth/standard length*100 (McLaren, 1958; Nilssen et al., 1997).

The following data on lungworm diagnosis and treatment was collected:

• *Clinical signs*; if the seal had clinical signs of a lungworm infection at intake.
• *Treatment for lungworms*; if the seal has received treatment against a lungworm infection (treatment = 1: no treatment = 0).
• *Confirmed diagnosis*; if the diagnosis of lungworm infection was confirmed by finding larvae in the faeces using the Baermann method, by (gross) necropsy, or was passively confirmed by the seal coughing up lungworms (yes = 1: no = 0).
• *Survival*; if the seal survived and was released back into the wild. Alternatively, if a seal had died or was euthanised (survival = 0: mortality = 1).

Additionally, stranded seals were assigned to one of four age class categories: pup, juvenile, subadult or adult, mainly based on body length according to research by McLaren (1993) and on the date of intake. From the date of intake an estimation of the seal's age could be made, since harbour seals have a defined birth season in June/July (Reijnders et al., 2010). However, McLaren (1993) does not distinguish pups and juveniles. In this study, pups were defined as animals that would not yet be weaned in the wild (approximately <3–4 weeks old) (Teilman and Galatius, 2018) and are categorised by the conditions in Table 1.

**Table 1:** Overview on age class categorisation of harbour seals. Four age class categories are distinguished: pup, juvenile, subadult, adult, based on McLaren (1993).

	Harbour seal age class categorisation
Pup	Weight <10 kg: pup OR Weight >10 kg: date of intake - i.e. +- 1–2 months from the start of the birth season in June are considered pups/juvenile, depending on: o For example: their clinical presentation and behaviour, for example presence of suckling behaviour; presence of (remains of) an umbilical cord or not fully emerged teeth.
Juvenile	Date of intake - >2 months after the from the start of the birth season: juvenile OR - + - 1–2 months from the start of the birth season: pups/juvenile, depending on: o For example: their clinical presentation and behaviour, for example absence of suckling behaviour and more defensive behaviour, such as biting and aggression towards caretakers. AND Age <1 year (seals less than one year after the end of the birth season).
Subadult	Date of intake: age >1 year AND Standard length (nose-tail; cm) - Male ≤ 142 cm - Female ≤ 129 cm
Adult	Standard length (nose-tail; cm) - Male > 142 cm - Female > 129 cm

### 2.2 Data analyses

The data were collected in an MS Excel™ spreadsheet and data analyses were performed with R version 3.6.0 (R Core Team, 2019) using the integrated development environment (IDE) of RStudio (RStudio Team, 2018). Mortality/survival was cross tabulated with the respective explanatory parameters. Univariable and multivariable logistic regression were used to examine the associations between the possible determinants and the outcome – mortality (binary) – resulting in odds ratios with 95% confidence intervals. For numerical parameters without available reference values (body weight; nose-tail length; nose-flipper length; axillary girth; body condition score I and body condition score II), data was categorised into two groups for each parameter based on the mean value; i.e. lower than the mean and higher than the mean. Associations with a p-value <0.2 in the univariable analysis were selected for the multivariable logistic regression analysis. First, a full model was created and subsequently backward model selection was used to derive the final model that best fits the data, based on the Akaike Information Criterion (AIC). The AIC value is a parameter that represents the likelihood of the statistical model and also accounts for the number of explanatory variables. The model with lowest AIC value is preferred to those with higher AIC values, as this is the most parsimonious model with sufficient fit (Akaike, 1973). Additionally, the determinants that were included in the full multivariable model were evaluated for collinearity by cross tabulating the determinants and assessing the relationship between those determinants using the Chi-squared test. Finally, in the backward elimination process of the modelling, confounding between variables were assessed using the change in parameter estimates which should not exceed 15%.

Seals with unknown survival status – i.e. seals that were moved to another rehabilitation centre or were still being cared for in the rehabilitation centre at the time of data analysis (n = 5) were kept in the descriptive analysis, but were omitted from further statistical analysis. The univariable analysis and multivariable model were conducted for juvenile harbour seals suffering from a lungworm infection. Clinical signs of a lungworm infection at intake and/or treatment for lungworm infection, either based on clinical signs or a confirmed diagnosis, were considered a proxy for lungworm infection. This resulted in not all seals included having a confirmed diagnosis. In the univariable logistic regression missing data was included in the cross tabulation but excluded for assessment of association and in multivariable logistic regression only complete cases were included (n = 134), excluding 16 seals.

## 3 Results

### 3.1 Descriptive analysis

**Fig. 1:**
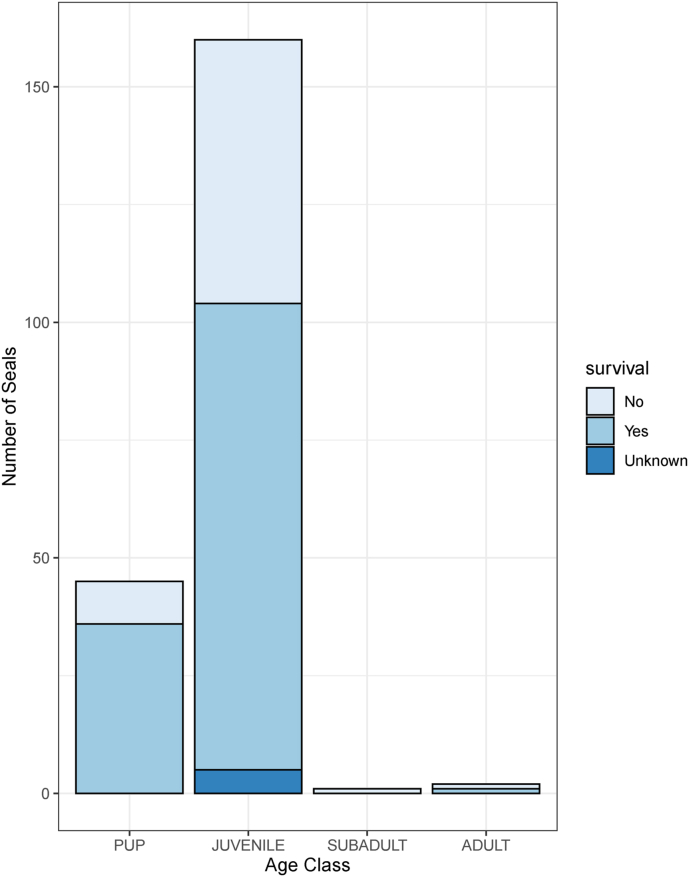
Harbour seal survival in the rehabilitation centre, see file ‘Fig. 1’ (p.8).

The main findings of the descriptive analysis are shown in Fig. 1. The majority of harbour seals in the rehabilitation centre were juveniles (n = 160; 76.9%) and their reported mortality in the rehabilitation centre was 36.1% (95%CI: 28.6; 44.2). This mortality rate is higher than in harbour seal pups, which was reported as 20%.

Fig. 1: Harbour seal survival in the rehabilitation centre per age group. Each bar represents the number of harbour seals in a specific age group rehabilitated in the centre between September 1, 2017 and August 31, 2019 (n = 208). The colour of each bar represents the survival within each group.

#### 3.1.1 Percentage of seals suffering from lungworm infections

Of the juvenile harbour seals (n = 160) 83.1% (n = 133) had clinical signs indicative of lungworm infection at intake. The total percentage of juvenile harbour seals treated for lungworm by the rehabilitation centre exceeded this at 91.8%. Among juvenile harbour seals suspected of a lungworm infection, 38.0% (n = 57) had a confirmed diagnosis. Other seals were diagnosed based on clinical signs. In seals suspected of a lungworm infection the mortality was reported as 35.3%.

### 3.2 Univariable analyses

Table 2 summarises the findings of the univariable analysis. The odds of mortality in seals that arrive at the rehabilitation centre with a higher body temperature (>37.9 °C) was significantly lower (Odds Ratio (OR) = 0.4; p = 0.03) than odds of mortality in seals with a normal body temperature at intake (Table 2).

**Table 2:** Number and percentages for mortality in juvenile harbour seals per determinant with odds ratio, 95% confidence interval and p-value analysed with univariable logistic regression (n = 150).

Parameters	Categories	Total	Mortality	Survived	OR	95% CI	p-value
		n (%)	n (%)	n (%)			
Total			53 (35.3)	97 (64.7)			
Sex	Male	75 (50.0)	25 (33.3)	50 (66.7)	Ref		
	Female	72 (48.0)	26 (36.1)	46 (63.9)	1.1	0.6–2.2	0.72
	Unknown	3 (2)	2 (66.7)	1 (33.3)			
Body temperature at intake (°C)	High (>37.9)	55 (36.7)	13 (23.6)	42 (76.4)	0.4	0.2–0.8	0.03
	Normal (36.5 - ≤ 37.9)	82 (54.7)	37 (45.1)	45 (54.9)	Ref		
	Low (<36.5)	7 (4.6)	2 (28.6)	5 (71.4)	0.5	0.07–2.4	
	Unknown	6 (4.0)	1 (16.7)	5 (83.3)			
Period of the year	August–December	73 (48.7)	20 (27.4)	53 (72.6)	0.5	0.3–1.0	0.05
	January–May	77 (51.3)	33 (42.9)	44 (57.1)	Ref		
Weight (kg)	<17.9	64 (42.6)	20 (31.2)	44 (68.8)	Ref		
	≥17.9	82 (54.7)	30 (36.6)	52 (63.4)	1.3	0.6–2.6	0.50
	Unknown	4 (2.7)	3 (75.0)	1 (25.0)			
Nose-tail length (Standard length; cm)	<94.2	72 (48.0)	23 (31.9)	49 (68.1)	Ref		
	≥94.2	69 (46.0)	27 (39.1)	42 (60.9)	1.4	0.7–2.8	0.37
	Unknown	9 (6.0)	3 (33.3)	6 (66.7)			
Nose-flipper length (cm)	<107.9	68 (45.3)	21 (30.9)	47 (69.1)	Ref		
	≥107.9	73 (48.7)	29 (39.7)	44 (60.3)	1.5	0.7–3.0	0.27
	Unknown	9 (6.0)	3 (33.3)	6 (66.7)			
Axillary girth (cm)	<68.8	71 (47.3)	22 (31.0)	49 (69.0)	Ref		
	≥68.8	64 (42.7)	27 (42.2)	37 (57.8)	1.6	0.8–3.3	0.18
	Unknown	15 (10.0)	4 (26.7)	11 (73.3)			
Body condition score I	<19.2	68 (45.3)	24 (35.3)	44 (64.7)	Ref		
	≥19.2	70 (46.7)	23 (32.9)	47 (67.1)	0.9	0.4–1.8	0.76
	Unknown	12 (8.0)	6 (50.0)	6 (50.0)			
Body condition score II	<73.2	67 (44.7)	22 (32.8)	45 (67.2)	Ref		
	≥73.2	67 (44.7)	26 (38.8)	41 (61.2)	1.3	0.6–2.6	0.47
	Unknown	16 (10.7)	5 (31.2)	11 (68.8)			
Previously rehabilitated	Yes	23 (15.3)	3 (13.0)	20 (87.0)	0.2	0.1–0.7	<0.01
	No	127 (84.7)	50 (39.4)	77 (60.6)			
Confirmed lungworm	Yes	57 (38.0)	23 (40.4)	34 (59.6)	Ref		
diagnosis	No	92 (61.3)	30 (32.6)	62 (67.4)	0.7	0.4–1.4	0.34
	Unknown	1 (0.7)	0 (0.0)	1 (100.0)			

AbbreviationsOR = odds ratio; CI = confidence interval; Ref = reference.

In addition, odds of mortality among seals that are in a rehabilitation centre for the first time is significantly higher (OR = 0.2; p < 0.01) compared to seals that have been in a rehabilitation centre before. No associations were found between mortality and the determinants: ‘sex’, ‘weight’, ‘nose-tail length’, ‘nose-flipper length’, ‘axillary girth’, ‘body condition score I’, ‘body condition score II’ and ‘confirmed diagnosis’.

### 3.3 Multivariable logistic regression analysis

In the final multivariable logistic regression four parameters were included: body temperature at intake, period of the year, axillary girth and previously rehabilitated. The final multivariable logistic regression model can be seen in Table 3. The odds of mortality was reported to be lower in seals with a high body temperature at intake (OR = 0.32) when compared to seals with a normal temperature, admitted from August–December (OR = 0.40) compared to January–May and in those that were previously rehabilitated (OR = 0.12) compared to seals not previously rehabilitated.

**Table 3:** Estimated adjusted odds ratio (OR) for mortality in juvenile harbour seals with lungworm, 95% confidence interval and p-value of the final multivariable logistic regression model (n = 134).

Parameters		95% CI		
	OR	Lower	Upper	p-value
Body temperature at intake				
High (>37.9)	0.32	0.14	0.74	0.01
Normal (36.5–37.9)	Ref			
Low (<36.5)	0.30	0.04	1.50	
Period of the year				
August–December	0.40	0.18	0.86	0.02
January–May	Ref			
Previously rehabilitated				
Yes	0.12	0.02	0.50	<0.01
No	Ref			
Full model for analysing mortality includes determinants: body temperature at intake; period of the year; axillary girth; and previously rehabilitated				

Abbreviations: OR = odds ratio; CI = confidence interval; Ref = reference.

## 4 Discussion

In order to ensure adequate decision making on whether or not a seal should be taken into rehabilitation, it is important to develop improved criteria for triaging seals. To achieve this goal, the aims of this study were: to estimate the proportion of lungworm infected juvenile harbour seals in a rehabilitation centre, to determine the survival rate of the lungworm infected juvenile harbour seals, and to study determinants of mortality in lungworm infected juvenile harbour seals. The results of this study demonstrate that the majority of all seals in the rehabilitation centre are juvenile harbour seals and that mortality in juvenile harbour seals is higher than in harbour seal pups. The final multivariable model revealed three determinants of mortality; the odds of mortality is lower in seals with an elevated body temperature at intake, which are taken in from August to December and in seals that were previously rehabilitated.

There are a number of possible causes that would explain the lower mortality among seals with an elevated body temperature compared to seals with a normal body temperature at intake. Firstly, seals with an elevated body temperature may potentially be treated differently by the staff working at the rehabilitation centre (an example of ‘confounding by treatment’). Seals with persistent elevated body temperatures are more likely to receive different supportive treatment, for example antibiotics or NSAIDs, compared to the standard treatment for lungworm. It is hypothesised that this additional treatment may increase the seal's probability of survival. However, as these treatments were variable and the data in the seal's electronic records was limited, statistical analysis could not be applied to this parameter. Furthermore, according to Hind and Gurney (1998), the normal body temperature of a seal is approximately 37.0 °C. However, currently no scientific evidence is available for the range of reference values for body temperature in harbour seals. Therefore, it is possible that the reference values for the normal body temperature used by the rehabilitation centre (36.5–37.9 °C) are inaccurate. Further research is required to provide reliable reference values for body temperature for seals (in rehabilitation).

A further finding of this study was that mortality rates for seals with an ID tag (or chip), identifying those that had been previously rehabilitated, was significantly lower than for seals taken in for the first time. One could hypothesize that seals that need to be taken into a rehabilitation centre again have poorer general health than seals taken into rehabilitation for the first time. An explanation could be that seals which were rehabilitated multiple times experience lower levels of stress due to habituation to the rehabilitation processes and facilities (Atkinson and Dierauf, 2018). For example, research by Trumble et al. (2013) identified decreased levels of average serum cortisol concentration in these seals during their stay in the rehabilitation centre. However, the use of serum cortisol concentration is often complicated by handling artefacts, due to the rapid release of cortisol when the seal is handled to obtain the blood samples (Crocker, 2018; Atkinson and Dierauf, 2018). Therefore, this study recommends that stress measures that demonstrate increased reliability for seals pre, during, and, where possible, post rehabilitation are established. These measures could also provide insight into the influence of stress on the survival of seals in rehabilitation. It can be reasoned that seals experiencing higher levels of stress have lower odds of survival, since prolonged or high levels of stress can have detrimental effects on the health of an animal (Atkinson and Dierauf, 2018). Non-invasive methods for collecting samples for measuring cortisol have been used in other (marine) mammals and should be considered, for instance faeces (Champagne et al., 2018), saliva (Ugaz et al., 2013) or hair (Bechshøft et al., 2012).

Furthermore, the results of this study demonstrate that the period of the year significantly influenced the odds of mortality. Mortality among juvenile harbour seals was significantly lower in seals that were admitted to the rehabilitation centre between August and December when compared to seals arriving between January and May. A possible explanation for this difference may be that seals perhaps require increased levels of energy for thermoregulation in the winter, thereby resulting in a different daily time distribution for certain activities. This is supported by the period from January to May coinciding with the lowest sea surface temperatures. In winter, more time is allocated to hunting and less time is spent resting (Harding et al., 2005). It can therefore be hypothesised that in seals suffering from a lungworm infection this altered time distribution and increased requirement for food can result in blubber loss, due to increased difficulty when hunting as a result of the effects of lungworm infection (Barnett and Bexton, 2016). Research has shown that in wild juvenile harbour seals, autumn weight can significantly impact survival. Seals with an autumn weight of 17 kg had a 0.63 chance of survival, while seals with a 32 kg autumn weight had a 0.96 chance of survival (Harding et al., 2005).

Axillary girth was initially included in the multivariable modelling. However, it was not retained in the final model. As no significant association between body weight and survival, body condition score and survival, as well as between length (nose-tail as well as nose-flipper length) and survival was identified, we suspect that a juvenile seal's size at the time of intake does not play an important role as a determinant of mortality in rehabilitation. However, caution must be taken as the results for the parameters nose-tail length (standard length), nose-flipper length and axillary girth may be influenced by sampling bias, as the quality of the measurements can be influenced by the experience of the seal handler and the experience of the person taking the measurements. Additionally, even with an experienced handler, the behaviour of the animal can also hinder taking (accurate) measurements, which may have accounted for some loss of data for the analyses.

We recognise that this study has a number of limitations that are relevant when interpreting the results and for future research. Although our study population is a census of all rehabilitated seal in the centre for a two year period, the data set is too limited to account for collinearity between determinants by including interaction terms in the modelling approach. For example, it can be reasoned that the parameter ‘period of the year’ and the parameter ‘previously rehabilitated’ are colinear due to the seasonal pattern of birth. Another limitation may be the potential categorisation bias despite the careful performance of age assessment. The data was categorised into four age groups: ‘pup’, ‘juvenile’, ‘subadult’ and ‘adult’ whereas, in the literature, seals are categorised in just three age groups. For example Brasseur (2018), divides the data into ‘young’, ‘subadult’ and ‘adult’ and McLaren (1993) describes ‘juvenile’, ‘subadult’ and ‘adult’. As the differentiation between pups and juveniles is practically relevant for the rehabilitation centre, it was deemed necessary to use four categories in this study, due to pups and juveniles requiring different care, for example using different husbandry and feeding protocols. This separation of pups and juveniles in harbour seals currently leaves some room for interpretation.

To our knowledge, no previous research has been performed on determinants of mortality of juvenile harbour seals (with lungworm) however, there are pre-existing studies of determinants of harbour seal pup survival. Witte et al. (2014) evaluated the association between blood gases and survival, but did not find a significant association. Additionally, Greig et al. (2010), discovered an association between two haematological parameters - decreased levels of platelets and decreased levels of protein - and survival, however the resulting models were poor fits to the data. Haematological parameters could not be evaluated in our study, as haematological examinations are not routinely performed at the rehabilitation centre and thus this data was not available.

In the rehabilitation centre the proportion of juvenile seals that were treated for lungworm infection was very high (91.8%). Clinical signs of a lungworm infection and/or treatment for lungworm infection, either based on confirmed or probable diagnosis, were considered as a proxy for lungworm infection in our study. However, in 61.3% (n = 92) of the seals, diagnostic testing was not conducted, with diagnosis based on clinical signs. Since the prevalence of lungworm infection in wild populations is high (89%) (Ulrich et al., 2016) we included all juvenile harbour seals that were treated for lungworm or had clinical signs of a lungworm infection at intake in the analyses. Currently, the only diagnostic test available for lungworm in seals is the Baermann method (Baermann, 1917). However, the results may demonstrate false negatives if the sample was taken during the pre-patent period, in which the animal can already have severe clinical signs (Barnett and Bexton, 2016). In research by Ulrich et al. (2015), a recombinant antigen-based enzyme-linked immunosorbent assay (ELISA) was described for lungworm detection in seals. The antigen ELISA has very high specificity (100%) and sensitivity (97.83%) but this diagnostic test is not commercially available. Such tests could possibly be used in future diagnostic testing to prevent animals from being treated unnecessarily.

We suggest the development of novel diagnostic protocols for live juvenile harbour seals suffering from lungworm infections to establish more accurate prognoses and to enable the administration of more selective therapies that demonstrate increased efficacy. One method to explore is diagnostic imaging by X-ray examination. X-ray, magnetic resonance imaging (MRI) and computed tomography (CT) scanning would allow examination of the lungs to reveal the presence and severity of lungworm infections and associated bronchopneumonia with increased objectivity and enabling the evaluation of other comorbidities.

In conclusion, three determinants of mortality were identified for lungworm infected juvenile harbour seals in a rehabilitation centre. These determinants are an important first step in providing seal rehabilitation staff with guidelines on whether or not a seal should be rehabilitated. Further research is needed to elucidate other determinants in a broader study population and evaluate alternative methods for determining the prognosis of seals in rehabilitation.

## Declaration of competing interest

The authors declare the following financial interests/personal relationships which may be considered as potential competing interests: Machteld Geut is a veterinarian employed by A Seal Rescue and Rehabilitation Centre. Marloes van Wijngaarden is a volunteer at A Seal Rescue and Rehabilitation Centre and a Master student of Veterinary Medicine (BSc).
